# High risk of metabolic syndrome among black South African women with severe mental illness

**DOI:** 10.4102/sajpsychiatry.v23i0.1089

**Published:** 2017-04-10

**Authors:** Shamima Saloojee, Jonathan K. Burns, Ayesha A. Motala

**Affiliations:** 1 Department of Psychiatry, Nelson R Mandela School of Medicine, University of KwaZulu-Natal, South Africa; 2 Department of Psychiatry, Institute of Health Research, University of Exeter, United Kingdom; 3 Department of Diabetes and Endocrinology, Nelson R Mandela School of Medicine, University of KwaZulu-Natal, South Africa

## Abstract

**Background:**

There is an increased prevalence of metabolic syndrome (MetS) in individuals with severe mental illness (SMI) globally. The prevalence of MetS is higher in black women compared to black men from South Africa.

**Aim:**

To compare the prevalence of MetS between black South African men and women with SMI taking antipsychotic medication. Further, this prevalence was compared to the prevalence in a matched control group of black South African men and women without SMI.

**Setting:**

A general hospital psychiatric unit.

**Methods:**

A cross-sectional study was undertaken to compare the prevalence of MetS in a group of multi-ethnic participants with SMI treated with antipsychotic medication and a matched control group without SMI, applying the 2009 Joint Interim Statement (JIS) criteria. Here, we included only the black African participants to compare MetS prevalence between men and women.

**Results:**

There were 232 participants in the group with SMI (male 155 and female 77) and without SMI (male 156 and female 76). The prevalence of MetS was more than three times higher in women with SMI compared to men with SMI (37.7% vs. 10.3%, *p* < 0.001). There was no significant difference in the prevalence of MetS in men or women between the groups with and without SMI. In multivariate logistic regression analysis, female gender (odds ratio [OR] 7.66), advancing age (OR 1.08) and longer duration of illness (OR = 1.15) were significant risk factors for MetS in SMI.

**Conclusion:**

In black South Africans with SMI on antipsychotic medication, there is a higher prevalence and risk for MetS in women compared to men.

## Introduction

From 1990 to 2013, there was an 81% increase in deaths from cardiovascular disease (CVD) in sub-Saharan Africa (SSA), with more women dying from CVD than men.^1^ Obesity, hypertension, dysglycemia and dyslipidaemia are individual as well as inter-related risk factors for CVD. When these risk factors are clustered together, they are known as the metabolic syndrome (MetS).^2^ MetS has clinical utility because its presence doubles the risk of developing CVD within the next 5–10 years,^2^ with reports of women from SSA having a higher age-adjusted standardised mortality from CVD than women from other developing and developed regions.^1^ Black women have a higher prevalence of obesity and MetS compared to black men in the general population of South Africa.^3^

The study of MetS in severe mental illness (SMI) is relevant because there is an increased prevalence of MetS in SMI.^4^ The cause of this increased prevalence is complex and multi-factorial,^5^ with a significant contribution from antipsychotic medication.^4^ From previous studies, there is a suggestion that women with SMI are at higher risk for MetS than men with SMI,^6,7,8^ possibly because of an increased susceptibility to the metabolic side effects of antipsychotics.^9^

An inconsistent association between race and ethnicity and the metabolic side effects of antipsychotics has been reported previously. For example, black patients taking olanzapine experienced more weight gain than white patients,^10^ but African Americans had a lower prevalence of MetS than white Americans in the CATIE trial.^6^

However, very little is known about gender differences in the prevalence of MetS among black patients with SMI taking antipsychotic medication from Africa.

We hypothesised that the prevalence of MetS will be higher in black women compared to black men with SMI taking antipsychotic medication from South Africa, because the prevalence of MetS is higher in black women compared to black men in the general population of South Africa^3^ and given that women with SMI and black patients may have an increased propensity to develop antipsychotic-induced adverse events.^9,10^

The aim of this study, therefore, was to compare the prevalence of MetS between black South African men and women with SMI taking antipsychotic medication. Further, the prevalence of MetS among black South African men and women with SMI taking antipsychotic medication was compared to the prevalence of MetS in a matched control group of black South African men and women without SMI. Identifying subgroups of patients with SMI who are at higher risk for MetS than others is important because the results can be used to guide focused screening and monitoring.

## Research methods and design

### Study design and setting

A group of black African, Indian, coloured (mixed ancestry) and white study participants with SMI taking antipsychotic medication were recruited from the psychiatric unit of a general hospital in Durban, South Africa, from February 2012 to December 2014 for a previously described cross-sectional study to investigate the prevalence of MetS in patients with SMI.^11^ That study comprised 276 inpatients and outpatients with SMI taking antipsychotic medication and a control group of 276 participants without SMI. The psychiatric unit has a 20 bed inpatient facility and provides a regional-level service for other district hospitals without psychiatrists; it also serves a primary health care function because it is close to a large informal settlement without community mental health clinics. In this analysis, we included only the black African participants to compare the prevalence of MetS between black African men and women

### Study population

Male and female patients who were aged 18–65 years, taking a first-generation antipsychotic (FGA) or second-generation antipsychotic (SGA) as monotherapy or polytherapy (combined with other psychotropic medication) for at least 3 months and who met Diagnostic and Statistical Manual of Mental Disorders (4th ed., text rev.; DSM-IV-TR; American Psychiatric Association, 2000) criteria for schizophrenia, bipolar 1 mood disorder or schizoaffective disorder were included in the study.^12^ Patients who lacked the capacity to provide informed consent and who were HIV positive or pregnant were excluded from the study.

Control participants were recruited by placing posters explaining the purpose and procedures for the study at the entrance, exit and other high-traffic areas in the hospital. Hospital staff, health science students and members of the public who were physically healthy and who had no lifetime diagnosis or treatment for a mental illness were invited to participate in the study. Control participants who were HIV positive or pregnant were excluded. Control participants were matched for age, gender and ethnicity with study participants.

### Data collection

Demographic and clinical data were obtained by the administration of a specially designed questionnaire and examination of clinical records. Age, gender, diagnosis of mental illness, duration of illness, antipsychotic prescriptions, a family and personal history of diabetes, hypertension or the use of lipid-lowering therapy were recorded on anonymised data sheets.

Weight in kilograms (kg) and height in centimetres (cm) were measured for calculation of the body mass index (BMI). BMI (kg/m^2^) was categorised as normal (< 25), overweight (25.0–29.9) and obese (> 30). Waist circumference was measured using a soft tape measure at the mid-point between the upper border of the iliac crest and the inferior margin of the last rib. Blood pressure was measured with the subject in the sitting position after a 10-min rest; two readings were taken with a minimum interval of 10 min, and the mean of the two readings was used to record the blood pressure. Venous blood sampling was performed after an overnight fast for plasma glucose and serum lipids (total cholesterol, total triglycerides, high-density lipoprotein [HDL] cholesterol and low-density lipoprotein [LDL] cholesterol). LDL cholesterol was calculated by the Freidewald formula.

The primary outcome measure was the prevalence of MetS, which was defined using the 2009 Joint Interim Statement (JIS) definition,^2^ which states that the presence of any three of the following five risk factors constitute the MetS: (1) increased waist circumference (waist circumference cut-off points used were: men ≥ 94 cm and women ≥ 80 cm); (2) elevated blood pressure: systolic > 130 mmHg (or on treatment) and diastolic > 85 mmHg (or on treatment); (3) elevated fasting plasma glucose ≥ 5.6 mmol/L (or on treatment); (4) elevated fasting triglycerides ≥ 1.7 mmol/L (or on treatment); and (5) reduced HDL cholesterol ≤ 1.0 mmol/L for men (or on treatment) and ≤ 1.3 mmol/L for women (or on treatment).

### Data analysis

Statistical analyses were performed using SAS, version 9.4 (SAS Institute, Cary, North Carolina). Descriptive statistics for demographic, behavioural, clinical and laboratory characteristics were expressed as means (± SD) for continuous variables and as percentages for categorical variables. The unpaired *t*-test was used to compare means of continuous variables, and Fisher’s exact test was used for the analysis of categorical data; unpaired *t*-tests or the Wilcoxon two-sample test was used for the analysis of continuous data. Stratified analyses were performed in order to determine whether age, duration of illness, diagnosis, cigarette smoking or antipsychotic medication were confounding the relationship between MetS and gender in patients with SMI. A logistic regression model, adjusting for age, diagnosis of mental illness, antipsychotic medication, duration of illness and cigarette smoking was fitted to the data to further examine the association between MetS and gender. All statistical tests were 2-sided and *p*-values < 0.05 were regarded as significant.

## Ethical considerations

This study was approved by the Biomedical Research Ethics Committee of the University of KwaZulu-Natal (BE 088/11). Written informed consent was obtained from all study and control participants in English or Isizulu.

### Results

Table 1 shows the characteristics of the study and control groups. The study and control group included 232 participants each. There was a greater proportion of men in both groups, 66.8% (*n* = 155) in the study group and 67.2% (*n* = 156) in the control group. Women were significantly older than men in the study (*p* = 0.003) and control group (*p* < 0.001). When compared to men with SMI, women were more likely to be diagnosed with schizoaffective disorder and bipolar disorder (*p* = 0.001), had a lower prevalence of cigarette smoking (5.2% vs. 61.3%) and a higher prevalence of total body obesity (BMI) (46.8% vs. 16.8%). Women with SMI had a higher mean waist circumference (*p* = 0.01), total serum cholesterol (*p* = 0.003) and LDL cholesterol level (*p* = 0.001) compared to men with SMI.

**TABLE 1:** Clinical and laboratory characteristics of the study group with severe mental illness and the control group.

Variable	Study group			Control group		
	Men (*n* = 155)	Women (*n* = 77)	*p*	Men (*n* = 156)	Women (*n* = 76)	*p*
**Age (years)**	**31.7 ± 10.7**	**37.2 ± 14.1**	**0.003**	**31.6 ± 9.9**	**38.0 ± 13.2**	**< 0.001**
**Age group (years) ([*n*] %)**			**< 0.001**			**0.001**
18–24	43 (27.7)	21 (27.3)		43 (27.6)	20 (26.3)	
25–34	68 (43.9)	15 (19.5)		69 (44.2)	16( 21.1)	
35–44	22 (14.2)	16 (20.8)		22 (14.1)	14 (18.4)	
45–54	15 (9.7)	13( 16.9)		15 (9.6)	15 (19.7)	
≥ 55	7 (4.5)	12 (15.6)		7 (4.5)	11 (14.5)	
**Diagnosis ([*n*] %)**			**0.001**			
Schizophrenia	126 (81.3)	47 (61)				
Schizoaffective disorder	7 (4.5)	15(19.5)				
Bipolar 1 disorder	22 (14.2)	15(19.5)				
**Duration of illness (years)**	**4.9 ± 5**	**4.7 ± 5.4**	**0.8**			
Cigarette smoking	95 (61.3)	4 (5.2)	< 0.001	75 (48.1)	1 (1.3)	< 0.001
Polytherapy	83 (53.5)	42 (54.5)	0.9			
FGA (monotherapy)	21 (13.5)	9 (11.7)	0.7			
SGA (monotherapy)	51 (32.9)	26 (33.7)	0.9			
BMI (kg/m^2^)	25.8 ± 7.0	30.9 ± 7.6	< 0.001	24.3 ± 4.7	33.2 ±8.6	< 0.001
**BMI category**			**< 0.001**			**< 0.001**
Normal: <2 5	91 (58.7)	20 (26 )		107 (68.6)	12 (15.8)	
Overweight: 25–29.9	38 (24.5)	21 (27.3)		32 (20.5)	17 (22.4)	
Obese: ³ 30	26 (16.8)	36 (46.8)		17 (10.9)	47 (61.8)	
Waist circumference (cm)	90.1 ± 13.2	95.8 ± 16.7	0.01	82.4 ± 11.6	95.9 ± 17.1	< 0.001
**Blood pressure (mmHg)**
Systolic	120.6 ± 14.4	123.7 ± 19.8	0.23	118.7 ± 13	124.9 ± 22.7	0.03
Diastolic	75.2 ± 10.3	76.4 ± 9.9	0.38	76.4 ± 10.9	75.7 ± 13.1	0.67
Fasting plasma glucose (mmol/L)	4.9 ± 0.8	5.2 ± 1.4	0.09	4.9 ± 0.8	5.3 ± 1.6	0.09
**Serum lipids (mmol/L)**
Total cholesterol	3.9 ± 1.0	4.3 ± 1.0	0.003	3.9 ± 0.8	4.2 ± 0.9	0.006
Total triglycerides	0.9 ± 0.6	1.0 ± 0.5	0.87	1 ± 0.7	0.8 ± 0.6	0.01
High-density lipoprotein cholesterol	1.2 ± 0.4	1.2 ± 0.3	0.99	1.2 ± 0.3	1.2 ± 0.3	0.8
Low-density lipoprotein cholesterol	2.3 ± 0.8	2.7 ± 0.9	0.001	2.2 ± 0.7	2.6 ± 0.8	< 0.001

BMI, body mass index; FGA, first-generation antipsychotic; SGA, second-generation antipsychotic.

The overall prevalence of MetS was more than three times higher in women with SMI compared to men with SMI (37.7% vs. 10.3%; *p* < 0.001). Regarding the individual MetS components, except for the systolic blood pressure and serum triglycerides, all the other components were significantly more prevalent in women with SMI compared to men with SMI (*p* < 0.05).

There was no significant difference in the overall prevalence and prevalence of the individual components of MetS between women with and without SMI. Similarly, there was no significant difference in the overall prevalence of MetS between men with and without SMI, but men with SMI had a higher prevalence of the elevated waist circumference and low HDL criterion than men without SMI (Table 2).

**TABLE 2:** Prevalence of metabolic syndrome and individual components in the study (*n* = 232) and control group (*n* = 232).

Variable	Study group		Control group	
	Men (*n* = 155)	Women (*n* = 77)	Men (*n* = 156)	Women (*n* = 76)
Metabolic syndrome	16 (10.3)	29 (37.7)***	15 (9.6)	26 (34.2)***
Elevated waist circumference (cm)	59 (38.1)†	64 (83.1)***	27 (17.3)	62 (81.6)***
**Elevated blood pressure (mmHg)**				
Systolic	23 (14.8)	16 (20.8)	29 (18.6)	23 (30.3)*
Diastolic	25 (16.1)	24 (31.2)**	31 (19.9)	24 (31.6)*
Elevated fasting plasma glucose (mmol/L)	16 (10.3)	16 (20.8)*	15 (9.6)	14 (18.4)
Elevated serum triglycerides (mmol/L)	14 (9.0)	9 (11.7)	19 (12.2)	4 (5.3)
Low HDL cholesterol (mmol/L)	65 (41.9)‡	56(72.7)***	39 (25)	52 (68.4)***
**Number of MetS criteria met**				
0	46 (29.7)	4 (5.2)	72 (46.2)	4 (5.3)
1	2 (1.3)	14 (18.2)	49 (31.4)	16 (21.1)
2	38 (24.5)	30 (39)	20 (12.8)	30 (39.5)
3	12 (7.7)	20 (26)	12 (7.7)	20 (26.3)
4	2 (1.3)	6 (7.8)	1 (0.6)	3 (4)
5	2 (1.3)	3 (3.9)	2 (1.3)	3 (4)

HDL, high-density lipoprotein.

Men versus women*p < 0.05; **p < 0.01; ***p < 0.001

† Elevated waist circumference (cm) in men in study group versus men in control group, *p* < 0.001; ‡, low HDL cholesterol (mmol/L) in men in study group versus men in control group, *p* < 0.01.

In stratified analysis, variables that were significant confounders of the relationship between MetS and gender in individuals with SMI were age (*p* = 0.003), duration of illness (*p* = 0.02), cigarette smoking (*p* < 0.001), schizophrenia (*p* < 0.001), schizoaffective disorder (*p* < 0.02), antipsychotic monotherapy (*p* < 0.001), antipsychotic polytherapy (*p* = 0.02) and SGA monotherapy (*p* = 0.005). Compared to men, the prevalence of MetS was higher in women taking both FGAs (monotherapy; 55.6% vs. 0%) and SGAs (monotherapy; 38.5% vs. 9.8%; *p* < 0.005) (Table 3).

**TABLE 3:** Stratified analysis of the factors associated with the prevalence of metabolic syndrome in men (*n* = 155) and women (*n* = 77) with severe mental illness.

Variable	Prevalence of metabolic syndrome		*p*
	Men (*n* = 155)	Women (*n* = 77)	
**Age group (years) ([*n*] %)**			
18–24	2/43 (4.7)	4/21 (19.1)	0.09
25–30	1/45 (2.2)	1/8 (12.5)	0.28
31–39	3/37 (8.1)	4/21 (19.1)	0.24
> 39	10/30 (33.3)	20/27 (74.1)	0.003
**Diagnosis ([*n*] %)**			
Schizophrenia	11/126 (8.7)	18/47 (38.3)	< 0.001
Bipolar 1 disorder	1/7 (14.3)	2/15 (13.3)	1
Schizoaffective disorder	4/22 (18.2)	9/15 (60.0)	0.02
**Duration of illness (years)**			
< 1	1/70 (1.4)	4/28 (14.3)	0.02
2–3	2/16 (12.5)	3/8 (37.5)	0.29
4–8	3/37 (8.1)	13/29 (44.8)	0.001
> 8	10/32 (31.3)	9/12 (75.0)	0.02
**Cigarette smoking**			
Yes	9/95 (9.5)	1/4 (25.0)	0.35
No	7/60 (11.7)	28/73 (38.4)	0.001
**Antipsychotic treatment**			
Monotherapy	5/72 (6.9)	15/35 (42.9)	< 0.001
Polytherapy	11/83 (13.3)	14/42 (33.3)	0.02
**FGA monotherapy**	**0/21 (0)**	**5/9 (55.6)**	**-**
SGA monotherapy	5/51 (9.8)	10 /26 (38.5)	0.005

FGA, first-generation antipsychotic; SGA, second-generation antipsychotic; SMI, severe mental illness.

Data are % (*N*/*n*). *N* –Subjects with MetS in sub – category; *n*, total number of subjects in sub – category.

In multivariate logistic regression analysis, after adjusting for the significant confounding variables, female gender (odds ratio (OR) 7.66, confidence interval (CI) 2.57–22.83, *p* < 0.001), advancing age (OR 1.08, CI 1.05–1.21, *p* < 0.001) and longer duration of illness (OR 1.15, CI 1.06–1.24, *p* = 0.001) remained significantly associated with MetS in SMI (Table 4).

**TABLE 4:** Multivariate logistic regression analysis of risk factors associated with MetS in black South Africans with severe mental illness.

Variable	OR† (95% CI)	*p*
Gender (female vs. male)	7.66 (2.57–22.83)	< 0.001
Age	1.08 (1.05–1.21)	< 0.001
Monotherapy (no vs. yes)	1.11 (1.45–2.77)	0.818
SGA monotherapy versus FGA monotherapy	1.46 (0.29 –7.32)	0.75
Duration of illness	1.15 (1.06– 1.24)	0.001
Diagnosis (bipolar mood disorder vs. schizoaffective disorder)	0.23 (0.04–1.25)	0.12
Diagnosis (schizophrenia vs. schizoaffective disorder)	0.63 (0.22–1.79)	0.61
Smoker (yes vs. no)	1.61 (0.53–4.96)	0.4

† adjusted odds ratio (OR).

CI, confidence interval; SGA, second-generation antipsychotic; FGA, first-generation antipsychotic; SMI, severe mental illness; MetS, metabolic syndrome.

## Discussion

The main finding of this study in black South Africans with SMI taking antipsychotic medication is a threefold higher overall prevalence of MetS, and an almost eightfold higher risk for MetS in black women with SMI compared to black men with SMI using JIS criteria. There was no difference in the overall prevalence of MetS between women with SMI and women without SMI, or men with SMI and men without SMI from a matched control group.

A higher prevalence of MetS in women with SMI has been reported in individual studies,^6^ but a recent meta-analysis that included men and women of different ethnicities from diverse geographical regions reported no gender differences in the prevalence of MetS.^4^ A higher prevalence in women was also reported from South Africa,^13^ but that study comprised patients of multiple ethnicities with no control group.

Regarding the individual criteria, a higher prevalence of the elevated waist circumference criterion of MetS among women with SMI is a consistent finding globally,^4^ but the difference in prevalence between men and women in this study (83.1% vs. 38.1%, *p* < 0.001) is a concern. However, the appropriateness of the waist circumference criteria currently used to diagnose MetS in black Africans has been called into question. There is a suggestion that gender differences in the prevalence of central obesity and MetS in black South African women may have been overestimated because of the use of inappropriate waist circumference cut offs for individuals of African descent.^3^ A report from South Africa has suggested that the current International Diabetes Federation (IDF) waist circumference cut offs in black African males be decreased by 8 cm and that for black African females be increased by 12 cm.^3^ The finding of a significantly higher prevalence of low HDL cholesterol among women with SMI compared to men with SMI in this study has been reported previously.^6^

In this study, women with SMI had an almost eightfold higher risk for MetS than men with SMI. A higher risk for MetS in women with SMI is in keeping with the findings of other reports from Turkey^7^ and Mexico.^8^ By contrast, Lee et al.^14^ reported a fourfold higher risk in Korean men with SMI. It is unlikely, therefore, that gender is a specific risk factor for MetS in SMI because there is considerable variability in the risk of MetS attributable to either gender. Furthermore, a direct comparison of the gender distribution of MetS in SMI in this study with that of studies from other regions is limited by the paucity of studies that included matched control groups from the same source population; many studies compared the prevalence and gender distribution of MetS in SMI with general population controls from large national samples.^4,6,7^

There was a significantly higher prevalence of MetS in women compared to men taking SGAs in the stratified analysis (*p* = 0.005), but this association did not remain significant in multivariate analysis. The prevalence of MetS was higher in women taking both FGAs and SGAs confirming the lack of a class effect for antipsychotic medication.^15^

Age is a known risk factor for MetS, and the cumulative exposure to antipsychotic medication and the effects of poor lifestyle choices over time is a key predictor of MetS in SMI.^4^ The younger mean age of the women in this study (37.2 years), compared to the mean age of women in studies from other regions, for example, the CATIE study (43.7 years) is a concern.^6^ Moreover, a significant proportion of women (27%) in this study were in the 18–24 age group underscoring the urgency for preventative programmes.

In this study, there was no significant difference in the prevalence and gender distribution of MetS between the study and control groups. Two studies, one from Venezuela^16^ and the other from Turkey,^7^ reported a similar overall prevalence of MetS in patients with SMI and a control group. However, only the Turkish study reported a higher prevalence in women, with the other reporting no significant gender difference.

The aetiology of CVD in SMI is known to be multi-factorial and the lack of a difference in the prevalence of MetS between the study and the control group in this study may be explained by common determinants of MetS in the two groups. It is possible that the role of antipsychotic medication in the aetiology of MetS in SMI may be exaggerated in the psychiatric literature^5,17^ and, key factors driving the increased prevalence of MetS in black women with SMI and black women in the general population in this study may be shared population-level risk factors for MetS, namely the rapid epidemiologic and nutritional transitions in the region,^3^ low activity levels, poor socioeconomic conditions, urbanisation^18^ and socio-cultural perceptions of overweight among black South African women.^19^ Our results therefore support antipsychotic independent mechanisms such as genetics, hormones, societal or life-style factors to account for the higher prevalence of obesity and MetS among black women with SMI.

Limitations of this study include the cross-sectional design which precluded causal associations and the lack of information regarding other recognised risk factors for MetS such as diet and physical activity. Furthermore, our results may not be generalisable and may be biased by the recruitment of participants and controls from a hospital setting only. Strengths include a within-study control group matched for age, gender as well as ethnicity and the inclusion of participants taking both FGAs and SGAs.

There is little doubt that CVD awareness, education and preventative interventions for women from high-income developed countries has resulted in substantially decreased mortality from CVD.^1^ This study provides evidence that can be used by public health policy makers and health care providers to institute similar heart health initiatives for women with SMI in South Africa. Metabolic screening and monitoring must be part of the standard of care for black women with SMI. Further research on MetS in South African patients with SMI is required utilizing larger sample sizes that include men and women of other ethnicities.

## Conclusion

This is the first study to draw attention to the higher prevalence and risk for MetS in black South African women with SMI on antipsychotic medication compared to men with SMI on antipsychotic medication. MetS is a risk factor for CVD, and unless corrective action is taken, many black women with SMI in South Africa could lose their lives each year, to what are essentially preventable conditions.
